# Targeting TANK-binding kinase 1 attenuates painful diabetic neuropathy via inhibiting microglia pyroptosis

**DOI:** 10.1186/s12964-024-01723-6

**Published:** 2024-07-19

**Authors:** Qinming Liao, Yimei Yang, Yilu Li, Jun Zhang, Keke Fan, Yihao Guo, Jun Chen, Yinhao Chen, Pian Zhu, Lijin Huang, Zhongjie Liu

**Affiliations:** 1 Department of Anesthesiology, Shenzhen Children’s Hospital, Shenzhen, 518000 Guangdong China; 2 Department of Neurosurgery, Guangdong Provincial Hospital of Chinese Medicine, Guangdong Provincial Academy of Chinese Medical Sciences, Guangzhou, 510030 Guangdong China; 3 Department of Neurosurgery, The Third Affiliated Hospital of Southern Medical University, Guangzhou, 510630 Guangdong China; 4 Department of Anesthesiology, Zhujiang Hospital of Southern Medical University, Guangzhou, 510220 Guangdong China; 5 Department of Neurosurgery, Dalang Hospital, Dongguan, 523775 Guangdong China; 6 Department of Anesthesiology, Zhongshan Hospital Affiliated to Fudan University, Shanghai, 200032 China

**Keywords:** Painful diabetic neuropathy, TBK1, Pyroptosis, Microglia, Amlexanox

## Abstract

**Background:**

Painful diabetic neuropathy (PDN) is closely linked to inflammation, which has been demonstrated to be associated with pyroptosis. Emerging evidence has implicated TANK-binding kinase 1 (TBK1) in various inflammatory diseases. However, it remains unknown whether activated TBK1 causes hyperalgesia via pyroptosis.

**Methods:**

PDN mice model of type 1 or type 2 diabetic was induced by C57BL/6J or BKS-DB mice with Lepr gene mutation. For type 2 diabetes PDN model, TBK1-siRNA, Caspase-1 inhibitor Ac-YVAD-cmk or TBK1 inhibitor amlexanox (AMX) were delivered by intrathecal injection or intragastric administration. The pain threshold and plantar skin blood perfusion were evaluated through animal experiments. The assessments of spinal cord, dorsal root ganglion, sciatic nerve, plantar skin and serum included western blotting, immunofluorescence, ELISA, and transmission electron microscopy.

**Results:**

In the PDN mouse model, we found that TBK1 was significantly activated in the spinal dorsal horn (SDH) and mainly located in microglia, and intrathecal injection of chemically modified TBK1-siRNA could improve hyperalgesia. Herein, we described the mechanism that TBK1 could activate the noncanonical nuclear factor κB (NF-κB) pathway, mediate the activation of NLRP3 inflammasome, trigger microglia pyroptosis, and ultimately induce PDN, which could be reversed following TBK1-siRNA injection. We also found that systemic administration of AMX, a TBK1 inhibitor, could effectively improve peripheral nerve injury. These results revealed the key role of TBK1 in PDN and that TBK1 inhibitor AMX could be a potential strategy for treating PDN.

**Conclusions:**

Our findings revealed a novel causal role of TBK1 in pathogenesis of PDN, which raises the possibility of applying amlexanox to selectively target TBK1 as a potential therapeutic strategy for PDN.

**Supplementary Information:**

The online version contains supplementary material available at 10.1186/s12964-024-01723-6.

## Introduction

The incidence of diabetes is rising and is estimated to be 12.2% in 2045 [1]. Non-insulin-dependent diabetes mellitus (type 2 diabetes mellitus, T2DM) is the prevalent form of diabetes, accounting for over 90% of cases globally [2]. Diabetic neuropathy has been reported to affect more than half of diabetic patients [3], with approximately 30% progressing to PDN [4], manifesting as allodynia, hyperalgesia, and spontaneous pain. Until now, treating PDN has been limited to hypoglycemic and analgesic strategies. Unfortunately, standardized blood glucose control could not significantly delay PDN [5].

Although the molecular events underlying the relationship between PDN and hyperalgesia remain uncertain, many studies have suggested an inflammatory link [6, 7]. Diabetic neuropathy exhibits the features of low-grade chronic inflammation [8]. Notably, diabetic rats with PDN had infiltration of inflammatory factors, and inflammation modulation ameliorated experimental PDN [9], suggesting that excessive inflammation is a key factor in PDN pathogenesis and thus may be a potential therapeutic intervention strategy.

TBK1, an abundant and ubiquitous serine/threonine-protein kinase of IκB kinase (IKK) family, has been widely reported to regulate innate immune responses against bacteria and viruses [10–12] and as a target for tumor treatment [13, 14]. Recent reports have stated that TBK1 dysfunction by inhibitor significantly played a protective role in inflammatory diseases, such as autoinflammatory arthritis, pathological maternal inflammation, and renal fibrosis [15–17], exposing the proinflammatory role of TBK1. Specifically, upon pathogen or damage-associated stimuli, TBK1 could be activated, mediating phosphorylation and nuclear translocation of interferon regulatory factor 3 (IRF3) and NF-κB, ultimately promoting inflammatory response [12]. Notably, activation of TBK1 and its upstream cyclic GMP-AMP synthase–stimulator of interferon gene (cGAS-STING) pathway has been confirmed in diabetic cardiomyopathy and pharmacologically inhibited STING could effectively reduce its inflammation-related damage and pyroptosis [18]. However, TBK1’s role in neuroinflammatory diseases has rarely been reported, and its potential mechanisms in PDN are yet to be elucidated.

A recent study found that pyroptosis promotes cytokine release and neurocognitive impairment in sepsis-associated encephalopathy [19], indicating a close relationship between pyroptosis and neuroinflammation. Gasdermin D (GSDMD) mediates an inflammatory form of regulated cell death known as pyroptosis that could be cleaved by active caspase-1, releasing the N-terminal domain of GSDMD (N-GSDMD). N-GSDMD binds to the inner plasma membrane and oligomerizes to form transmembrane pores, disrupting local osmotic gradients and causing localized cellular swelling and rupture to release cellular contents [19–24]. The major cell type of pyroptosis in the nervous system is microglia pyroptosis. Although GSDMD-induced microglia pyroptosis has been implicated in neuroinflammatory-related diseases, such as spinal cord injury [25] and subarachnoid hemorrhage [26], the underlying mechanism of this process in PDN has not been explored. Therefore, whether there is a correlation between TBK1, microglia pyroptosis, and PDN is yet to be determined.

This study demonstrated that TBK1 and GSDMD are upregulated in the SDH of type 1 diabetes mellitus (T1DM) and T2DM-related PDN mouse models. We found that TBK1 knockdown via chemically modified siRNA suppressed the microglia pyroptosis in the SDH and ameliorated hyperalgesia of PDN mice. Furthermore, we demonstrated that AMX, a clinical oral drug and an inhibitor of TBK1, suppressed spinal cord inflammation and improved peripheral nerve injury, ultimately relieving PDN.

## Materials and methods

### Animals

Male BKS-DB/Nju (db/db) mice (6–7 weeks old, a type of mutant mouse of the Lepr gene, strain number: T002407), age-matched littermate non-diabetic WT mice, and male C57BL/6J mice (4–5 weeks old) were obtained from GemPharmate Co., Ltd. (Jiangsu, China). Mice were kept in specific pathogen-free (SPF) rooms with a constant temperature of 24 ± 0.5 °C and 12-hour light/dark cycles, with free access to food and water. All experimental procedures were performed in accordance with the guidelines of the International Association for the Study of Pain and approved by the Animal Ethics Committee of Zhujiang Hospital of Southern Medical University (Ethics: LAEC-2021-003 and LAEC-2023-025, Guangzhou, China).

### Establishment of the PDN model

To establish the T1DM model, male C57BL/6 J mice were injected intraperitoneally with 100 mg/kg streptozotocin (STZ, Selleckchem, Cat#S1312) after four weeks of a high-fat and high-sugar diet, and continued to maintain the diet for 6 weeks after STZ injection. The model was deemed successful when fasting blood glucose, measured weekly, exceeded 11.1 mmol/L twice in a row, combined with a decrease in serum insulin. The db/db mice aged seven weeks were used to develop the T2DM model, often with markedly elevated blood glucose around seven weeks old.

To establish and validate the PDN model of two types of diabetes, paw withdrawal threshold (PWT) for mechanical allodynia and paw withdrawal latency (PWL) for thermal hyperalgesia were measured in all mice. Mice were tested at fixed times and in a fixed sequence of cage positions.

Specifically, mice were acclimated to the testing environment three days prior to baseline testing. To test mechanical allodynia sensitivity PWT, a series of Von Frey filaments (North Coast, USA) with a force ranging of 0.02–1.4 g were employed to stimulate the plantar skin of the left hind paw, and we determined the 50% PWT by Dixon’s up-down method [27, 28]. To examine the thermal sensitivity, PWL was measured with Hargreaves plantar apparatus (Ugo Basile biological instruments, Italy) following published methods [29]. The mice’s left hind paw was stimulated three times at five-minute intervals, and the average reading was calculated for each mouse.

### Knockdown of TBK1 in SDH with chemically modified siRNA technique

Specific chemically modified siRNA-oligo was applied to knockdown the TBK1 expression. Three siRNAs with 2’-O-Methyl oligonucleotide modification targeting mouse *Tbk1* gene were designed and synthesized by Genepharma (Shanghai, China). The sequences of three siRNAs were as follows: siRNA1 (sense: 5’-GGAAGUGUCCAAGUAUCAATT-3’; antisense: 5’-UUGAUACUUGGACACUUCCTT-3’). siRNA2 (sense: 5’-GCGUAUGGACUUCCAGAAUTT-3’; antisense: 5’-AUUCUGGAAGUCCAUACGCTT-3’); siRNA3 (sense: 5’-GCUCCUGUCUGAUAUCCUATT-3’; antisense: 5’-UAGGAUAUCAGACAGGAGCTT-3’); three siRNAs were transfected into the ND7/23 cells respectively using lipofectamine 3000 (Invitrogen, Carlsbad, CA). The expression level of TBK1 was examined using Western blot. According to the sequence of the siRNA3 with the remarkable knockdown effect, chemically modified scrambled non-targeting siRNA (siScr, sense: 5’-GUCUCACCUCCGGUAUAUUTT-3’; antisense: 5’-AAUAUACCGGAGGUGAGACTT-3’) with the same percentage of GC and AT but with no corresponding sequence was used as a negative control siRNA.

Chemically modified siRNA-oligo was administered by intrathecal injection after establishing the PDN mice model. The intrathecal injection was performed on mice anesthetized with isoflurane, as previously reported [30]. Briefly, mice were restrained with the left hand. Using a microsyringe with a 30G needle, we injected into the subarachnoid space between the L_5_ and L_6_ vertebrae of the mice. A tail-flick response confirmed proper needle entry. The siRNA injection was performed once every three days for three weeks (seven times). The dosage of siRNA was 2 µg (5 µL) per injection.

### Drug administration

Caspase-1 inhibitor Ac-YVAD-cmk (Selleckchem, Cat#S9727) was administered by intrathecal injection in the same manner as siRNA-oligo. Ac-YVAD-cmk (10 nmol per mouse) in 4% dimethyl sulfoxide (DMSO) in a total volume of 5 µL was injected every two days for three weeks.

AMX (Selleckchem, Cat#S3648) was dissolved in Sodium carboxymethyl cellulose (CMC-Na, Selleckchem, Cat#S6703), and administered by daily oral gavage (5, 25, or 100 mg/kg) for four weeks after establishing the PDN model. The control group was given CMC-Na solution without AMX.

### Experimental groups

Mice were grouped according to the numerical order from small to large that generated randomly using the random number generator function of SPSS 26 software (Inc., Chicago, IL, USA).

To investigate the hyperalgesia and expression of key proteins such as TBK1 in T1DM and T2DM-related PDN mice, C57BL/6J and db/db mice were randomly divided into two groups respectively: Vehicle (*n* = 5) and PDN (STZ) (*n* = 5); WT (*n* = 5) and PDN (DB) (*n* = 5). To test the appropriate dosage of siRNA, non-diabetic WT mice were divided into 3 groups: Vehicle (*n* = 6), si-TBK1 2 µg (*n* = 6) and si-TBK1 10 µg (*n* = 6). To explore the curative effect of TBK1-siRNA and Ac-YVAD-cmk (Caspase-1 inhibitor) on PDN mice, mice were divided into four groups: WT + si-Scr (*n* = 7), PDN + si-Scr (*n* = 6), PDN + si-TBK1(*n* = 6) and PDN + Ac-YVAD-cmk (*n* = 7). To observe the effect of amlexanox (TBK1 inhibitor) on the hyperalgesia in PDN mice, mice were divided into six groups: WT (veh) (*n* = 6), WT + AMX (25 mg/kg) (*n* = 6), PDN (Veh) (*n* = 6), PDN + AMX (5 mg/kg) (*n* = 6), PDN + AMX (25 mg/kg) (*n* = 6), PDN + AMX (100 mg/kg) (*n* = 6). To further evaluate the impact of amlexanox on protein expression, we focused on the intervention group administered amlexanox 25 mg/kg that mentioned above, and three groups were labeled: WT (*n* = 6), PDN (*n* = 6), and PDN + AMX (*n* = 6).

To establish the PDN model, 54 age-matched db/db mice, 44 littermate non-diabetic WT mice, and 10 C57BL/6J mice were used in this study. Six db/db and two WT mice were excluded (irritability, dead and unsuccessful PDN model).

The researcher was blinded to the grouping of mice during the experimental procedures. The experimental results were analyzed by a second person who was blinded to the group assignment to avoid any bias.

### Cell lines

ND7/23 cells were obtained from the Cell Bank of Chinese Academy of Sciences (Shanghai, China) and used for siRNA transfection experiments. Twenty-four hours after the cells were seeded, the culture medium was replaced with Opti-MEM medium (Invitrogen, Cat#31,985,070). Lipo3000 (Invitrogen, Cat#L3000001) and chemically modified siRNA were mixed, and left to stand at room temperature for 10 min to form a transfection complex. Add the complex to the cell culture dish and culture it in a 37 °C, 5% CO_2_ incubator. After 6–8 h, the culture medium was replaced and the transfection rate was evaluated under a fluorescence microscope; after 36 h, the cells were collected for protein determination.

### Primary DRG neuronal culture and cell treatment

As previously described, dorsal root ganglia (DRG) neuronal cell culture was prepared from mice [31]. Briefly, the L_4_-L_6_ segmental DRG was collected, followed by adding 1 mL of digestive enzymes (0.1% collagen type I − 0.3% dispase type II) at 37 °C for about 30 min. The isolated DRG neurons were plated in 0.1 mg/ml PLD + 5 mg/ml laminin-coated 24 well plates with cell crawling sheet (d = 14 mm, biosharp) and cultured in a complete medium containing DMEM/F12 (25 mM glucose), 1× B27, and 20 mg/ml nerve growth factor at 37 °C and 5% CO_2_ for 24 h. To investigate the neurite outgrowth assessment of DRG neurons, cells were treated with primary antibody beta-tubulin (Abcam, Cat#ab52623, 1:500), then immunofluorescence (IF) staining was performed and finally examined under confocal microscopy (Nikon). The longest neurite was measured using Image-Pro Plus 6.0 software.

### Measurement of blood flow of plantar skin

A laser speckle flow imager system (Simopto, China) was employed to observe blood perfusion in plantar skin. After the mice were anesthetized, bilateral posterior planar skin was exposed and observed for 5 min until stable. The image capture parameters of the device were set as follows: the exposure time was set to 10 ms, and the frame rate was one image per 10 s. The perfusion units (PU) in the selected region of interest (ROI) were recorded, and the average perfusion value within 100 s was calculated.

### ELISA assay

Mice’s serum was used to measure serum insulin, IFN-β and TNF-α. Mouse INS ELISA Kit (MEIMIAN, Cat#MM-0579M1), Mouse IFN-β ELISA Kit (Neobioscience, Cat#EMC016.96), and Mouse TNF-α ELISA Kit (Neobioscience, Cat#EMC102a.96) were used.

### Western blotting

The spinal cord tissue of the L_4_-L_6_ segment was extracted and homogenized in lysis buffer. Protein samples were analyzed by Western blot and performed according to published protocols [30]. The primary antibodies included anti-TBK1 (Cell Signaling Technology, Cat#38,066), anti-Phospo-TBK1 (Cell Signaling Technology, Cat#5483), anti-NF-κB p65 (Cell Signaling Technology, Cat#8242), anti-Phospho-NF-κB p65 (Cell Signaling Technology, Cat#3033), anti-NLRP3 (Immunoway, Cat#YT5382), anti-pro-Caspase-1 (Immunoway, Cat#YT5743), anti-cleaved Caspase-1 p20 (Cell Signaling Technology, Cat#89,332), anti-ASC (Abmart, Cat#PA7120), anti- IL-1β (Immunoway, Cat#YM4682), anti-GSDMD n-ternal (Immunoway, Cat#YT7991), anti-c-Fos (Cell Signaling Technology, Cat#2250), anti-Iba1 (Abcam, Cat#ab283319) and anti-TNF-α (Abcam, Cat#ab215188) (all diluted 1:1000).

### Immunofluorescent staining

Immunofluorescent staining was done following the previous methods [26]. Briefly, the L_4_-L_6_ segmental spinal cord sections (10 μm) were incubated with primary antibody including anti-TBK1 (Santa, Cat#sc-398,366, 1:300), anti-Iba1 (Abcam, Cat#ab178847, 1:300), anti-GFAP (Cell Signaling Technology, Cat#80,788, 1:500), anti-NeuN (Abcam, Cat#ab177487, 1:500), anti-GSDMD n-ternal (Immunoway, Cat# YT7991, 1:200), anti-NeuN (Sigma, Cat#MAB37,1:500), anti-GFAP (Cell Signaling Technology, Cat#3670, 1:500),anti-Iba1(Abmart, Cat#TU308558, 1:300), anti-Phospho-NF-κB p65 (Cell Signaling Technology, Cat#3033, 1:800), anti-c-Fos (Cell Signaling Technology, Cat#2250, 1:1500) and anti-TNF-α (Abcam, Cat#ab215188, 1:200) at 4 ℃ overnight. Slides were viewed and captured using a Nikon fluorescence microscope (TI2-E, Japan) after incubating tissues with corresponding fluorescent secondary antibodies and DAPI.

To analyze the intraepidermal nerve fiber density (IENFD), left posterior planar skin cryosections (50 μm thick) were stained with antibody against protein gene product 9.5 (anti-PGP9.5, Abcam, Cat#ab108986,1:250). Representative images of intraepidermal nerve fibers were captured with a Nikon fluorescence microscope (TI2-E, Japan). The number of nerve fibers crossing the dermal-epidermal junction was measured, and IENFD was defined by the number of fibers per centimeter of epidermal length.

### Morphology of sciatic nerve and DRG

The left sciatic nerve, or DRG, was prefixed in 2.5% neutral glutaraldehyde, postfixed in 1% osmic acid, and dehydrated in ascending ethanol concentrations. They were then infiltrated with Epon812 resin and set in resin blocks in an oven at 60 °C for 48 h for the transmission electron microscope. Axon diameter of myelinated and unmyelinated fibers and myelin sheath thickness were measured. Myelinated fibers were also evaluated using the g-ratio, calculated as a square root of the ratio of the axon to fiber area. G-ratio reflects the relative thickness of the myelin sheath, with its increase serving as a measure of myelin thinning. Images were digitalized with Image-Pro Plus 6.0 software.

### Statistical analysis

SPSS 26 (Inc., Chicago, IL, USA) was used to statistical analysis. Data were evaluated for normality. ANCOVA was used to analyze the data of plantar and Von Frey tests and weight and blood glucose over time. Comparison between multi-groups was determined by one-way ANOVA and followed by the Tukey test for two groups’ comparison within the multi-group unless otherwise specified. Student’s t-test was employed for two-group comparisons. Data that did not conform to a normal distribution, nonparametric test, and Kruskal-Wallis test were used for multi-group comparisons. For all panels, data are shown as mean ± SEM. In all cases, *P* < 0.05 was considered statistically significant.

The sample size was calculated using GPower3.1 software based on the behavioral data of the pilot study. A total of five times of repeated measurements of PWT and PWL data before and after the intervention were used for statistical testing using repeated measures analysis of variance (ANCOVA), setting parameter α err prob to 0.05 and Power (1-β err prob) to 0.8. The sample size for each group was five.

## Results

### Overactivation of TBK1 and upregulation of inflammatory response and pyroptosis in the SDH of mice with PDN

To determine whether TBK1 is implicated in T1DM and T2DM, C57BL/6 J mice were injected intraperitoneally with 100 mg/kg streptozotocin (STZ) to establish T1DM, and db/db mice aged seven weeks were used to establish T2DM. The PDN (STZ) group exhibited increased blood glucose and body weight, whereas serum insulin was decreased as compared to the Vehicle group, indicating that mice of the PDN (STZ) group were typical T1DM (Fig. S1A-C). T2DM mice were also established based on a significant increase in body weight, blood glucose, and serum insulin of the PDN (DB) group compared to the WT group (Fig. S1D-F). Simultaneously, we measured the paw withdrawal threshold (PWT) and paw withdrawal latency (PWL). T1DM mice exhibited a significant reduction in PWT and PWL two and four weeks after STZ injection, respectively, exhibiting a continuous downward trend (Fig. S1G-H). T2DM mice mainly displayed reduced PWT and PWL in the ninth week, maintained at a low level (Fig. S1I-J). At 13 weeks of age in the PDN(DB) group and 6 weeks post STZ injection in the PDN(STZ) group, we collected samples and further examined the expression and activation of TBK1 in the L_4_-L_6_ segment of the spinal cord. The activation of TBK1 was significantly upregulated in both types of diabetic mice (Fig. S1K-N), indicating a potential relationship between TBK1 and PDN.

The SDH is the primary center for transmitting and regulating pain signals and is crucial for pain perception in PDN. We demonstrated excessive expression of TBK1 in the SDH of the PDN (STZ) group or PDN (DB) group compared with the Vehicle or WT group, consistent with elevated TBK1 expression, as shown by IF staining (Fig. S2A-D).

To assess the inflammation level, the immunostaining signal of TNF-α was measured and found to be significantly overexpressed in both types of the PDN models, compared to the Vehicle or WT groups (Fig. S2E-H), exposing upregulated inflammatory reactions in SDH of PDN mice. However, to determine whether the high levels of inflammation could induce inflammation-associated cell death termed pyroptosis, we focused on the expression of GSDMD protein. According to the IF results, GSDMD was upregulated in the SDH of both types of PDN mice compared to the Vehicle or WT groups (Fig. S2I-L). Thus, the presence of pyroptosis in the SDH of PDN mice was determined.

### Silencing TBK1 with chemically modified siRNA or suppression of pyroptosis alleviated hyperalgesia of PDN mice

**Fig. 1:**
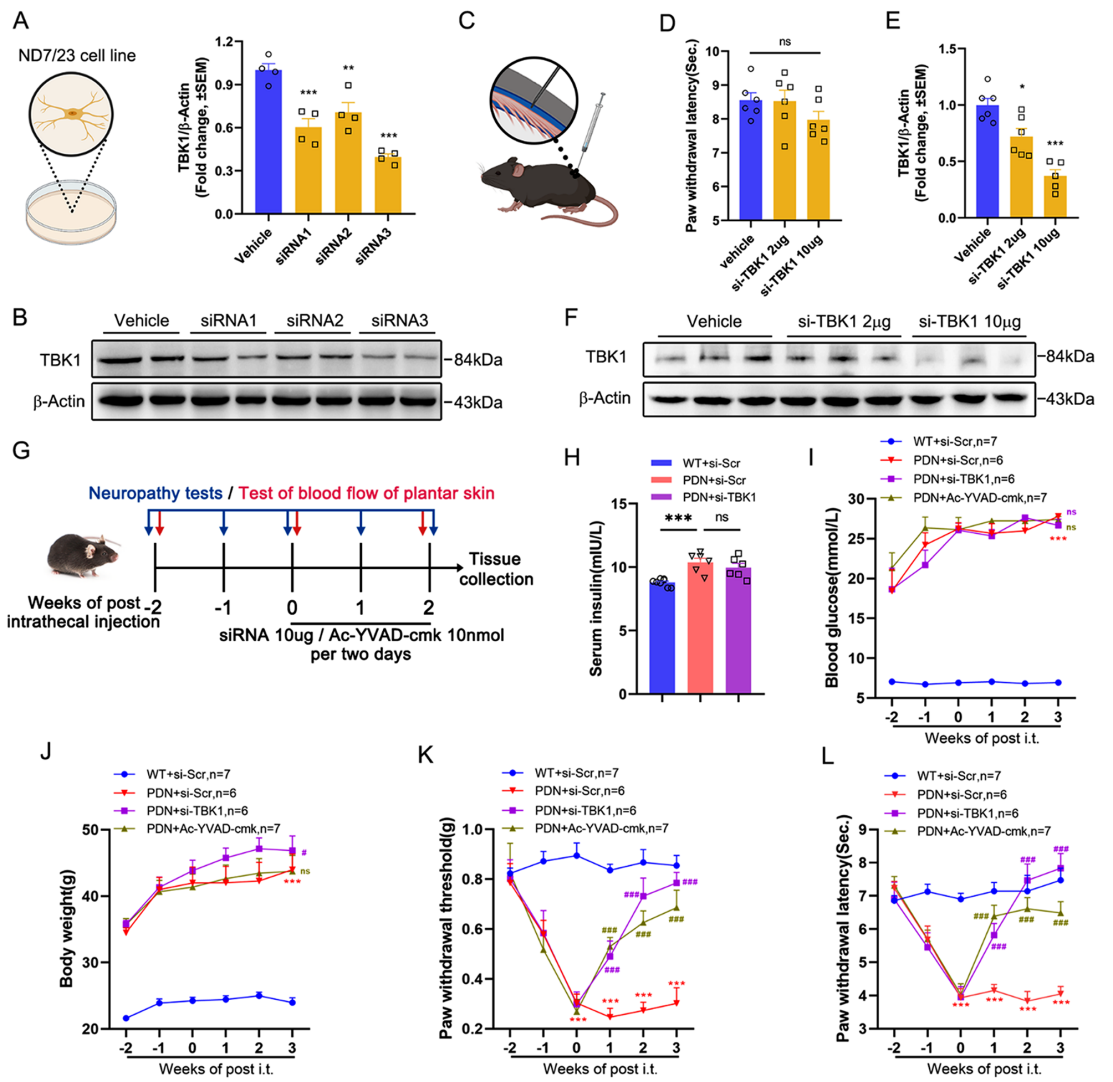
Improvement of hyperalgesia in PDN mice after intrathecal injection of screened TBK1-siRNA and Ac-YVAD-cmk. **A-B** Expression level of TBK1 in ND7/23 cells transfected with three types of siRNAs. **P < 0.01, ***P < 0.001 vs. Vehicle group. **C-F** Comparison of PWL and expression level of TBK1 in L_4_-L_6_ spinal cord after intrathecal injection of different doses of si-TBK1 (siRNA3) in WT mice. ns *P* > 0.05, *P < 0.05, ***P < 0.001 vs. Vehicle group. **G** Experimental diagram showing the timeline of neuropathy tests, blood flow of plantar skin test, and injection of siRNA and Ac-YVAD-cmk. **H** ELISA analysis of serum insulin in mice following siRNA injection. ns *P* > 0.05, ***P < 0.001. **I-L** Blood glucose and body weight were also measured. The established mechanical allodynia and thermal hyperalgesia were attenuated after the intrathecal injection of si-TBK1 and Ac-YVAD-cmk. ns *P* > 0.05 vs. PDN + si-Scr group; ***P < 0.001 vs. WT + si-Scr group; ###*P* < 0.001 vs. PDN + si-Scr group

Given the potential role of TBK1 in PDN, we first screened siRNA3, a chemically modified siRNA that could significantly silence TBK1 through cell-line experiments (Fig. 1A and B). Furthermore, animal experiments confirmed an appropriate intervention dose of 10 µg per mouse without exhibiting adverse behavioral effects on wild-type mice (Fig. 1C-F). Then, the T2DM mice model was established, and intrathecal injection of 10 µg si-TBK1 (siRNA3) or caspase-1 inhibitor Ac-YVAD-cmk 10 nmol per mouse was given every two days for three weeks. Notably, silencing TBK1 or inhibiting caspase-1 in spinal cord tissue did not affect insulin, blood glucose, and body weight levels (Fig. 1H-J). As for neuropathy tests, our results showed that intrathecal injection of si-TBK1 could reverse the reduced PWT (Fig. 1K) and PWL (Fig. 1L), revealing a promoting role of TBK1 in PDN. In addition, inhibiting caspase-1 could lessen pyroptosis, partially alleviating hyperalgesia (Fig. 1K-L), suggesting that caspase-1-dependent pyroptosis also plays a vital role in PDN.

### Microglia were the main localized cells of TBK1; silencing TBK1 could reduce the aggregation of microglia in PDN mice

**Fig. 2:**
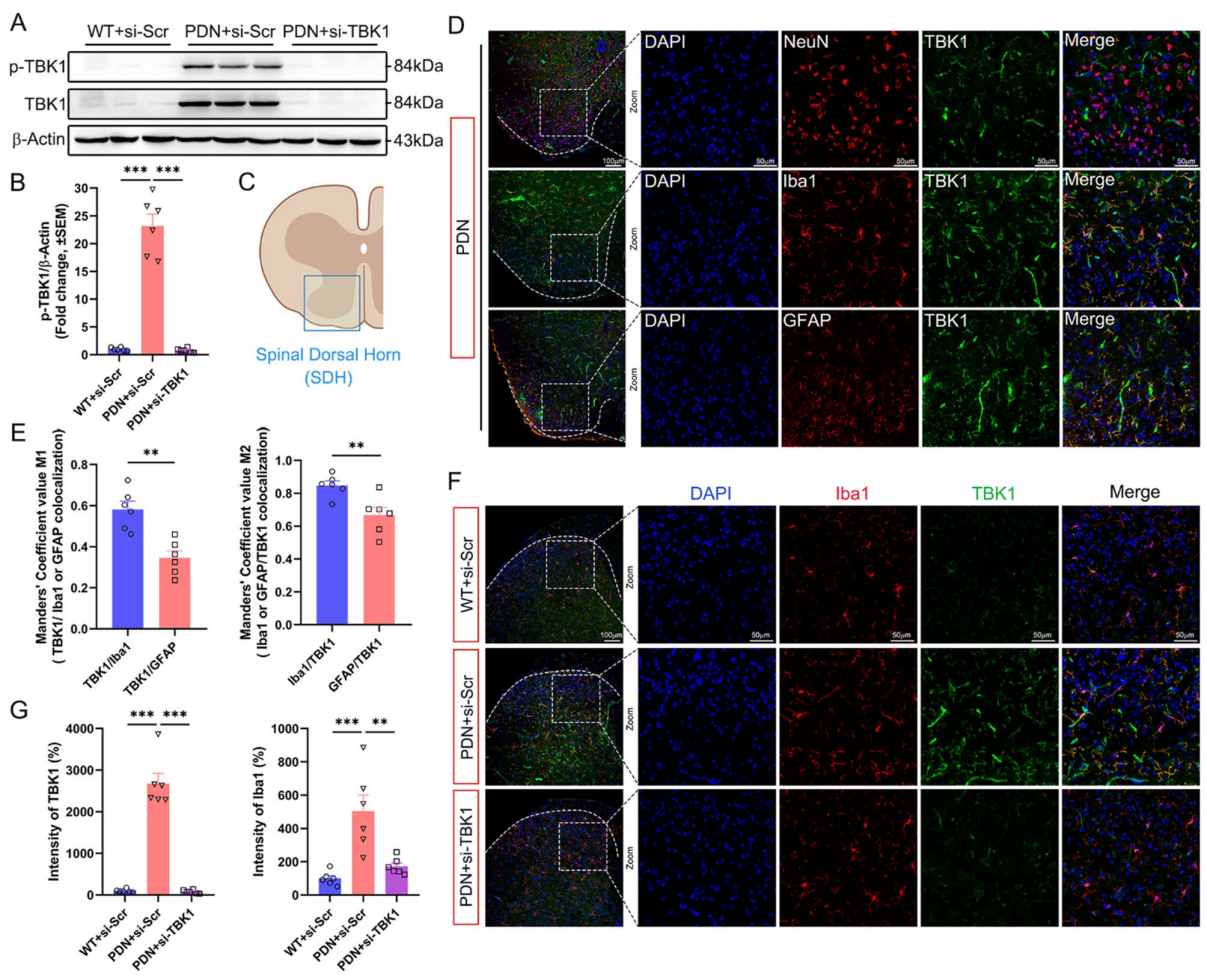
Active TBK1 was overexpressed in PDN mice and mainly localized in microglia in the spinal dorsal horn (SDH), knocking down TBK1 reduced microglial aggregation. **A-B** Western blot analysis of the expression and activation of TBK1 in the L_4_-L_6_ segment of the spinal cord of mice. **C** Schematic diagram of SDH. **D-E** Co-localization analysis of TBK1 in the L_4_-L_6_ segment of SDH in PDN mice showed that TBK1 was mainly localized in microglia (Iba1). **F-G** Change of fluorescence intensity of TBK1 and aggregation of microglia (Iba1) in SDH after siRNA injection in different groups. Data are shown as mean ± SEM.**P < 0.01, ***P < 0.001

Following the animal experiment, we obtained mice’s L_4_-L_6_ segment of the SDH to detect the expression and activation of TBK1 by Western blot (WB) analysis. The expression of TBK1 and Phospo-TBK1 (p-TBK1) in the PDN + si-Scr group was significantly higher than in the WT + si-Scr group, and si-TBK1 could effectively inhibit the expression and activation of TBK1 in the PND + si-TBK1 group (Fig. 2A-B).

Immunofluorescent co-staining revealed that TBK1 was primarily expressed in microglia (Iba1^+^) and astrocytes (GFAP^+^) and rarely in neurons (NeuN^+^) in the SDH of PDN mice (Fig. 2D). To elucidate whether microglia or astrocytes play a more significant role in TBK1 expression, we quantified it using Manders’ coefficient, a measure of the fraction of one structure to another [32]. The Manders’ coefficient M1 indicated the fraction of the TBK1 with Iba1 or GFAP, whereas the Manders’ coefficient M2 specified the fraction of Iba1 or GFAP with TBK1 (Fig. 2E). These results illustrated that microglia play a major role in the expression of TBK1.

The immunofluorescent co-staining of Iba1 and TBK1 confirmed that the trend of the fluorescence intensity of TBK1 in different groups followed the same trend as the WB analysis. We also found that Iba1 was highly expressed in the PDN + si-Scr group, whereas in the PDN + si-TBK1 group, the expression of Iba1 was downregulated, and its co-localization with TBK1 was reduced (Fig. 2F-G).

### Intrathecal injection of si-TBK1 alleviated microglial pyroptosis

**Fig. 3:**
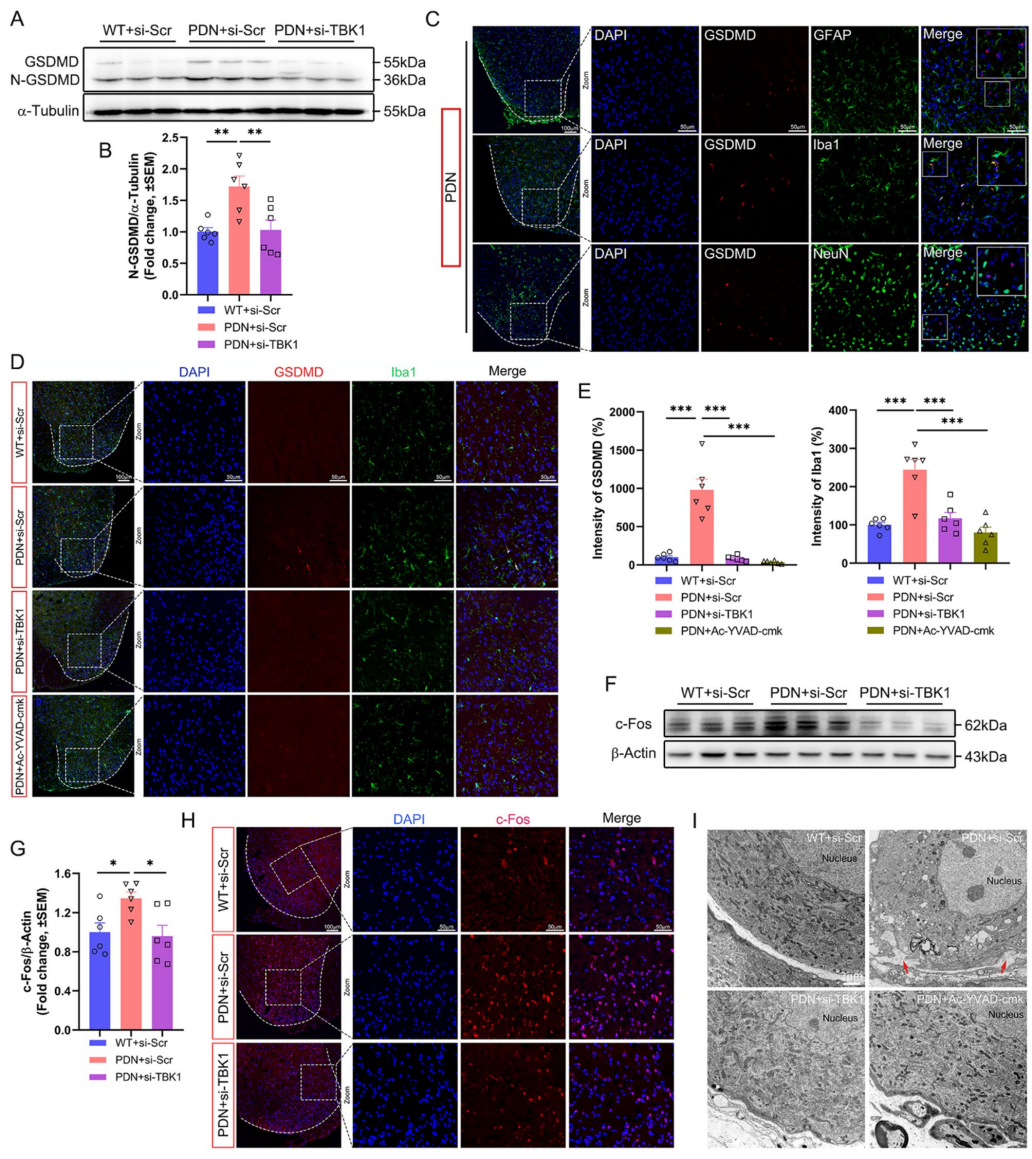
Silencing TBK1 alleviated pyroptosis and suppressed neuronal activity. **A-B** Western blot images and analysis showing GSDMD and N-GSDMD in the spinal cord. **C** Cell localization of GSDMD in the SDH of PDN mice showed that GSDMD was mainly localized in the microglia (Iba1+) but rarely in astrocytes (GFAP+) and neurons (NeuN+). **D-E** Change of fluorescence intensity of GSDMD and Iba1 in the SDH after siRNA or Ac-YVAD-cmk injection. **F-G** Immunoblot analysis of c-Fos expression levels in the spinal cord. **H** Visualization of c-Fos (red) expressing in the SDH through immunofluorescence staining. **I** Representative transmission electron micrographs of neurons in dorsal root ganglion (DRG). Red arrowhead: membrane pores. Data are shown as mean ± SEM. **P < 0.01, ***P < 0.001

The WB results revealed that compared to the WT + si-Scr group, N-GSDMD was increased in the PDN + si-Scr group and decreased in the PDN + si-TBK1 group (Fig. 3A-B). IF staining displayed that GSDMD was predominantly localized in Iba1^+^ microglia in the SDH (Fig. 3C). Moreover, microglia (Iba1+) were found aggregated in the SDH of the PDN + si-Scr group with highly expressed GSDMD. Microglial aggregation was downregulated, and the GSDMD expression was reduced after silencing TBK1 or inhibiting caspase-1 (Fig. 3D-E). In this case, we confirmed the effect of TBK1 on nervous system pain at the molecular level by measuring the expression of c-Fos, a neuronal activity marker (Fig. 3F-H). Interestingly, it was observed using a transmission electron microscopy that GSDMD membrane pore was increased in neurons of DRG in the PDN + si-Scr group compared with the WT + si-Scr group, inconsistent with the findings in the SDH; however, TBK1-siRNA or Ac-YVAD-cmk injection mitigated this trend (Fig. 3I).

**Fig. 4:**
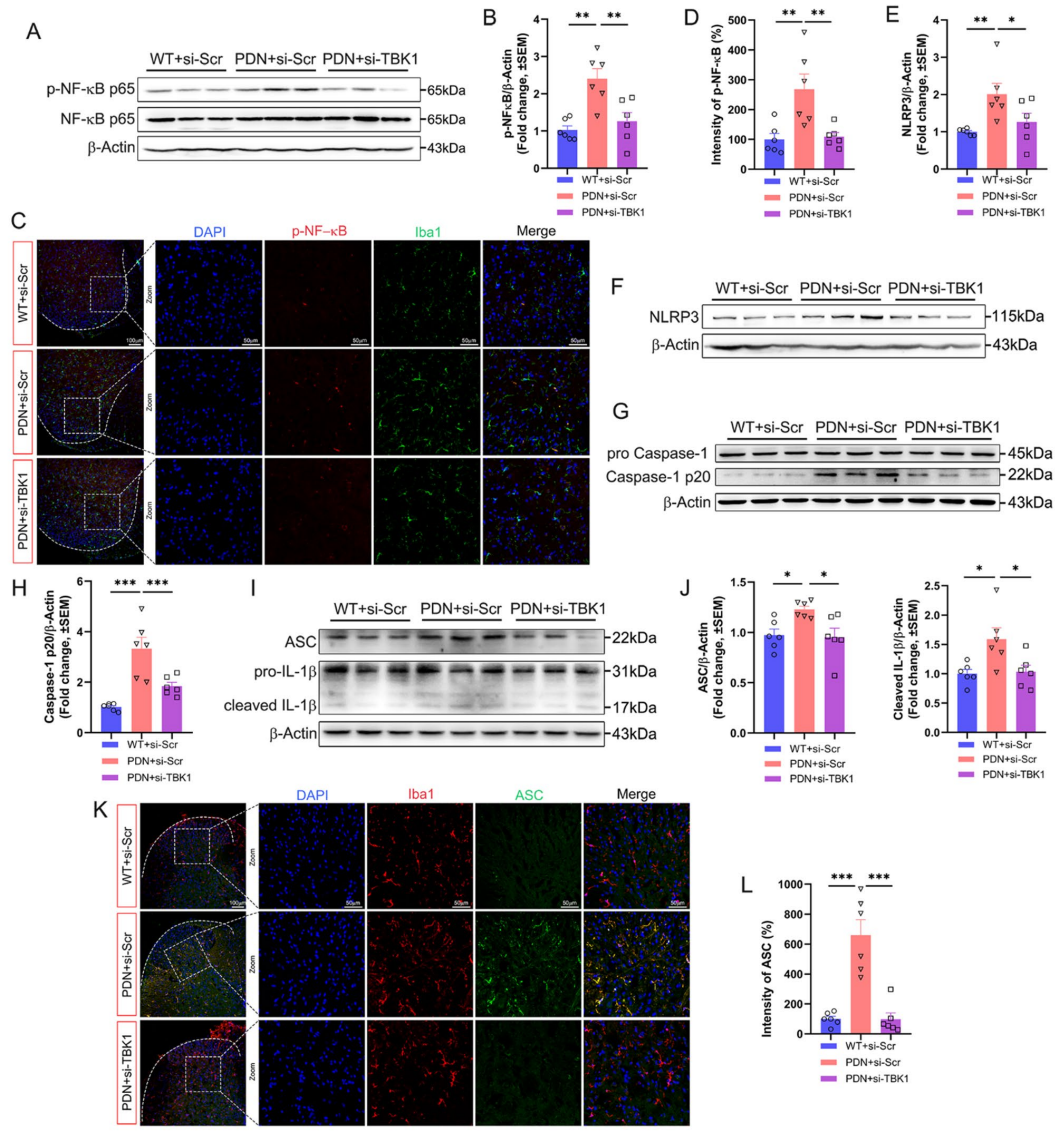
Inhibition of TBK1 suppressed the expression of NF-κB, NLRP3 inflammasome and IL-1β. **A-B** Immunoblot analysis of NF-κB expression levels in the spinal cord. **C-D** Double immunofluorescence staining of p-NF-κB and Iba1 in different groups. **E-F** Immunoblot analysis of NLRP3 expression levels in the spinal cord. **G-H** Immunoblot analysis of caspase-1 expression levels in the spinal cord. **I-J** Immunoblot analysis of ASC and IL-1β expression levels in the spinal cord. **K-L** Double immunofluorescence staining of ASC and Iba1 in different groups. Data are shown as mean ± SEM. *P < 0.05, **P < 0.01

As a downstream molecule of TBK1, NF-κB is closely related to NLRP3 [33], and NLRP3 inflammasome activation is involved in cell pyroptosis [34]. Here, we demonstrated that NF-κB was activated in the PDN + si-Scr group and inhibiting TBK1 could effectively reduce the NF-κB expression (Fig. 4A-B), the fluorescence intensity of p-NF-κB showed the same tendency as WB, and co-localization with microglia (Fig. 4C-D). We further clarified the role of NLRP3/ASC/caspase-1 activation as a bridge between TBK1 and membrane pore formation. WB analysis found that the increased TBK1 activation was accompanied by elevated levels of NLRP3 inflammasome (NLRP3, ASC, and caspase-1) and cleaved IL-1β. However, the increments of these proteins were repressed by TBK1-siRNA treatment (Fig. 4E-J). The fluorescence intensity of ASC showed the same tendency as WB, and co-localization with microglia (Fig. 4K-L).

### TBK1 knockdown of the spinal cord partially ameliorated peripheral nerve injury

**Fig. 5:**
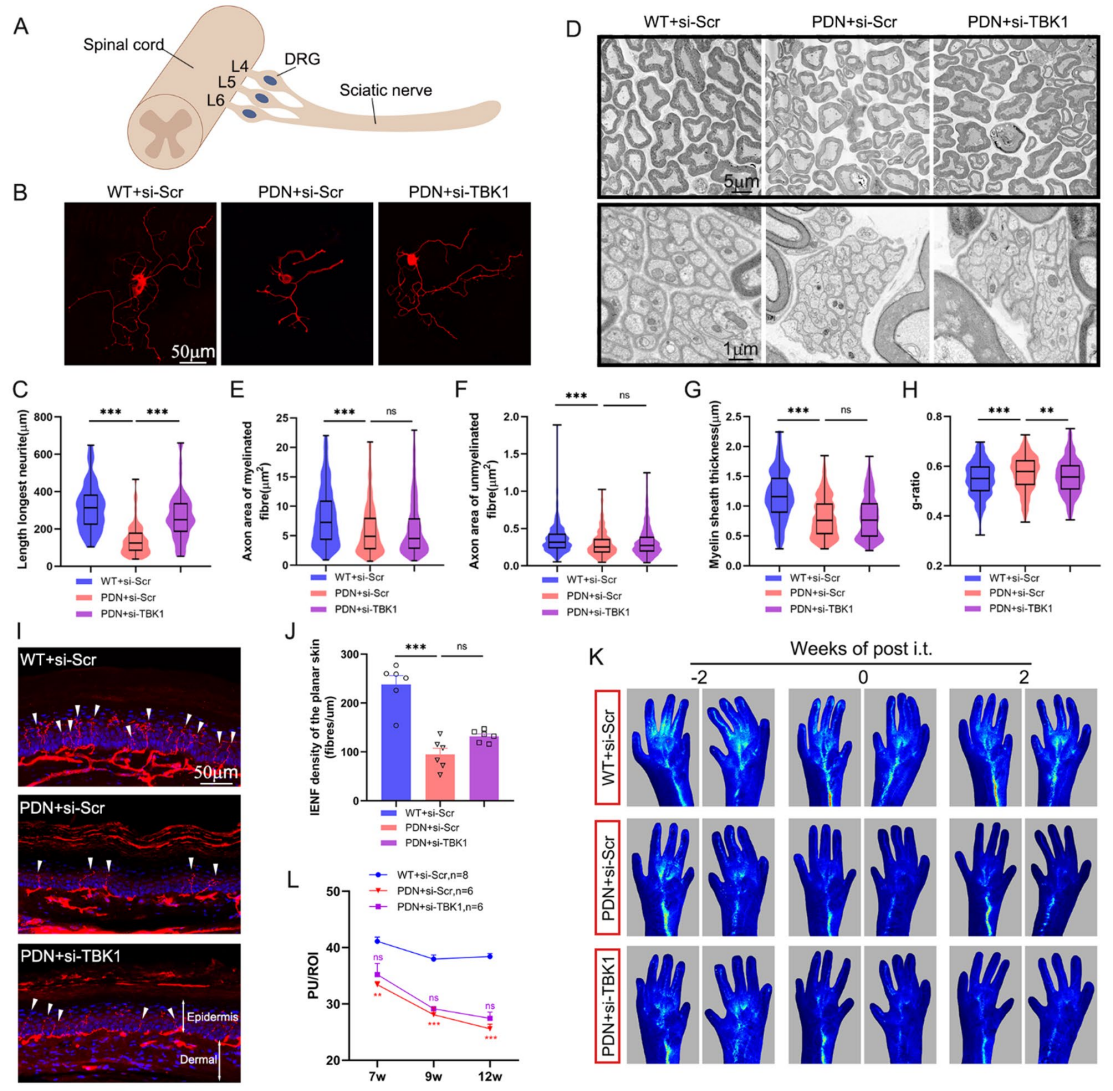
The effect of knocking down TBK1 of the L_4_-L_6_ segment of the spinal cord on peripheral nerve injury. **A** Schematic diagram of spinal cord segment of knocking down TBK1 in relation to lumbar DRGs (L_4_, L_5_, L_6_) and sciatic nerve. **B-C** Beta-tubulin immunofluorescent staining was used to observe the longest neurite length of primary neuronal cells of DRG. **D-H** Representative transmission electron micrographs of histomorphological changes in sciatic nerves. The upper images largely displayed myelinated fibers, while the lower images showed unmyelinated fibers. The scatter plots depict the changes in the axon area of ​​myelinated **E** and unmyelinated fibers **F** of the sciatic nerve and the myelin sheath thickness **G** and g-ratio **H** of the myelinated fibers. ns *P* > 0.05, **P < 0.01, ***P < 0.001. **I-J** Representative images exhibiting PGP9.5 immunoreactive intraepidermal nerve fibers (red, arrows) in different groups of hind plantar paw skin. Histogram representing the quantitative data of the nerve fiber density under various conditions. ns *P* > 0.05, ***P < 0.001. **K-L** Representative laser speckle flow images of plantar skin of mice. Blood flow of plantar skin was measured, and perfusion signals were presented in different colors: low to high as blue-green-red. The quantitative analysis of blood flow based on laser speckle flow imaging. ns *P* > 0.05 vs. PDN + si-Scr group; **P < 0.01, ***P < 0.001 vs. WT + si-Scr group

To understand the effects of TBK1 on peripheral nerves and microvessels, we measured various indicators from DRG to sciatic nerve and to plantar skin. Following the primary culture of DRG neurons, βIII-tubulin antibody was employed for IF staining. The neurite growth of the PDN + si-Scr group was inhibited compared with that of the WT + si-Scr group, and the use of TBK1-siRNA rescued the ability of DRG neurons to extend neurites in vitro (Fig. 5B-C).

Moreover, sciatic nerve analysis found that the PDN + si-Scr group exhibited a substantial reduction of axon area of myelinated and unmyelinated fibers and myelin sheath thickness and increased g-ratio compared to WT mice. However, intrathecal injection of TBK1-siRNA could reduce g-ratio; notably, it had no significant effect on axon area and myelin sheath thickness (Fig. 5D-H). However, the g-ratio can be affected by many factors. In addition to the axon area and myelin sheath thickness, it also has a certain correlation with the morphology of the fiber. When the absolute values of the axon area and myelin sheath thickness of the two groups are consistent, the rounder and more regular the fiber, the higher the g-ratio. So, our data showed that even after TBK1-siRNA injection, the sciatic nerve did not have a significant change in morphology.

Finally, we analyzed the plantar skin from two aspects: IENFD (Fig. 5I-J) and blood perfusion (Fig. 5K-L). The results found that these two indicators were significantly lowered in the PDN + si-Scr group, while intrathecal injection of TBK1-siRNA did not significantly reverse this outcome.

### Intragastric injection of TBK1 inhibitor AMX relieved neuropathic pain of PDN mice

**Fig. 6:**
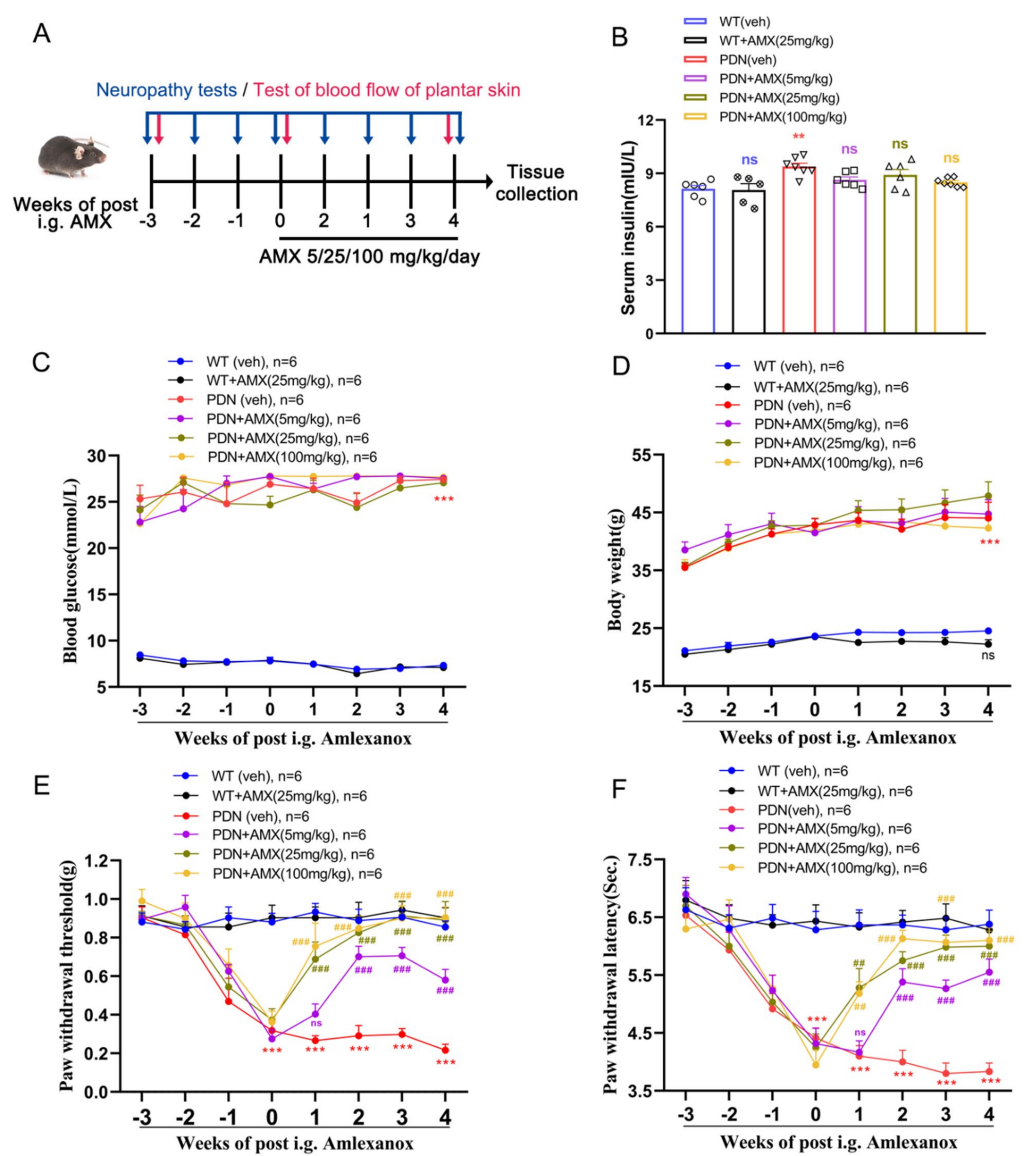
The role of TBK1 inhibitor, amlexanox, on hyperalgesia and metabolism. **A** Experimental diagram showing the timeline of neuropathy tests, blood flow of plantar skin test, and intragastric administration of different doses of amlexanox. **B** ELISA analysis of serum insulin in different groups. ns *P* > 0.05, **P < 0.01 vs. WT (veh) group. **C-F** Blood glucose and body weight measured in different groups. The effect of amlexanox on established mechanical allodynia and thermal hyperalgesia of PDN mice. ***P < 0.001 vs. WT (veh) group; ns *P* > 0.05, ##*P* < 0.01, ###*P* < 0.001 vs. PDN (veh) group

From the above-mentioned interventions in the spinal cord, we observed TBK1’s role in the central nervous system, having limited peripheral effects. We identified a systemic and clinically available drug, AMX, commonly used to inhibit TBK1 [35]. We used a dose gradient of 5/25/100 mg/kg to intervene. Unlike intrathecal injection of TBK1-siRNA, systemic application of AMX improved insulin resistance in PDN mice (Fig. 6B); however, it exhibited no significant changes in blood glucose and body weight (Fig. 6C-D). Notably, different doses of AMX could alleviate hyperalgesia in PDN mice, especially at doses of 25 or 100 mg/kg, having no adverse reaction in WT mice (Fig. 6E-F).

### Systemic application of AMX downregulated inflammatory response and ameliorated peripheral nerve and microvascular function of PDN mice

We further investigated the mice treated with 25 mg/kg of AMX. According to the WB and IF analyses of the spinal cord, AMX could effectively inhibit TBK1 activation (Fig. S3A-B). The change trend in TBK1 staining using IF was consistent with that of p-TBK1 in the SDH (Fig. S3C).

The increased Iba1 protein expression in the spinal cord of PDN mice indicated microglial cell aggregation and AMX could suppress this trend. (Fig. S3D-E). AMX systemic application reversed the high expression of inflammatory factor, TNF-α in the spinal cord of PDN mice (Fig. S3F-G) and TNF-α and IFN-β in serum (Fig. S3H). Similarly, IF was used to stain the TNF-α in the SDH, and results were consistent with the above-mentioned WB analysis (Fig. S3I).

**Fig. 7:**
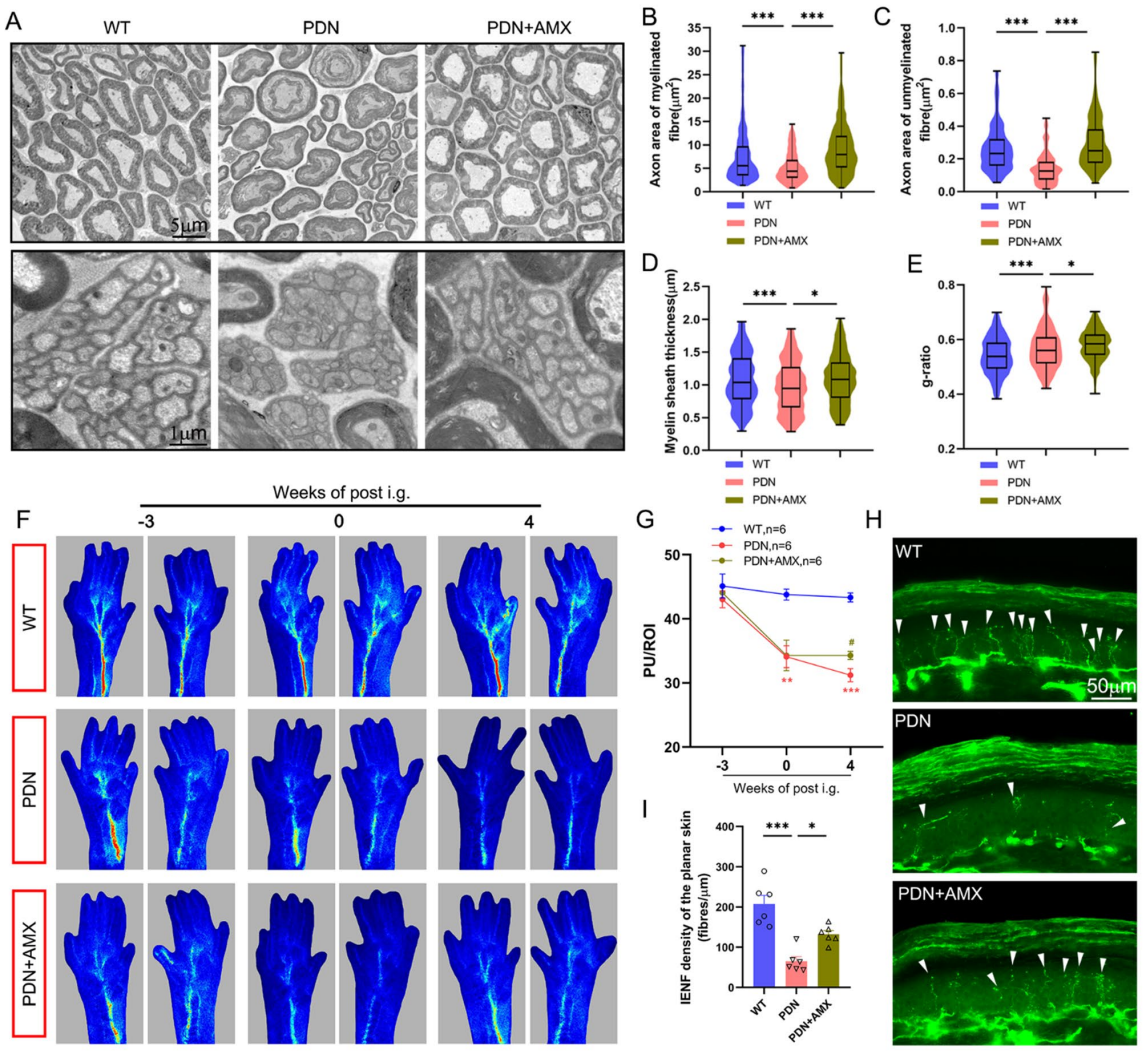
Effect of amlexanox on peripheral nerve injury of PDN mice. **A-E** Representative transmission electron micrographs of histomorphological changes of sciatic nerves. The upper images chiefly depict myelinated fibers, while the lower images present unmyelinated fibers. The scatter plots of the changes in the axon area of ​​myelinated **B** and unmyelinated fibers **C** of the sciatic nerve of PDN mice and the myelin sheath thickness **D** and g-ratio **E** of the myelinated fibers. *P < 0.05, ***P < 0.001. **F-G** Representative laser speckle flow images of plantar skin of mice and quantitative analysis of blood flow based on laser speckle flow imaging. **P < 0.01, ***P < 0.001 vs. WT group; #*P* < 0.05 vs. PDN group. **H-I** Representative images displaying PGP9.5 immunoreactive intraepidermal nerve fibers (green, arrows) in the hind plantar paw skin in different groups. Histogram representing the quantitative data of the nerve fiber density. * *P* < 0.05, ***P < 0.001

Knowing that TBK1 knockdown targeting the spinal cord was ineffective in improving sciatic nerve and plantar lesions, we pondered whether the results would differ with systemic administration of TBK1 inhibitors. AMX could effectively suppress the reduction of axon area of myelinated and unmyelinated fibers of the sciatic nerve in PDN mice, inhibiting thinning of the myelin sheath thickness of myelinated fibers (Fig. 7A-D). Notably, the g-ratio of PDN + AMX group continued to increase (Fig. 7E), combined with the normal recovery of myelin sheath thickness. Thus, we concluded that the reason was the significant increase in the area of the axon.

After analyzing the blood perfusion of the plantar skin, we found that blood perfusion of the PDN + AMX group did not deteriorate further after four weeks of AMX treatment; however, there was still a gap compared to the WT group (Fig. 7F-G). In addition, protein gene product 9.5 (PGP 9.5) IF staining was performed on the plantar skin, and it was noted that the IENFD of the mice in the PDN + AMX group was significantly higher than that of the PDN group (Fig. 7H-I).

**Fig. 8:**
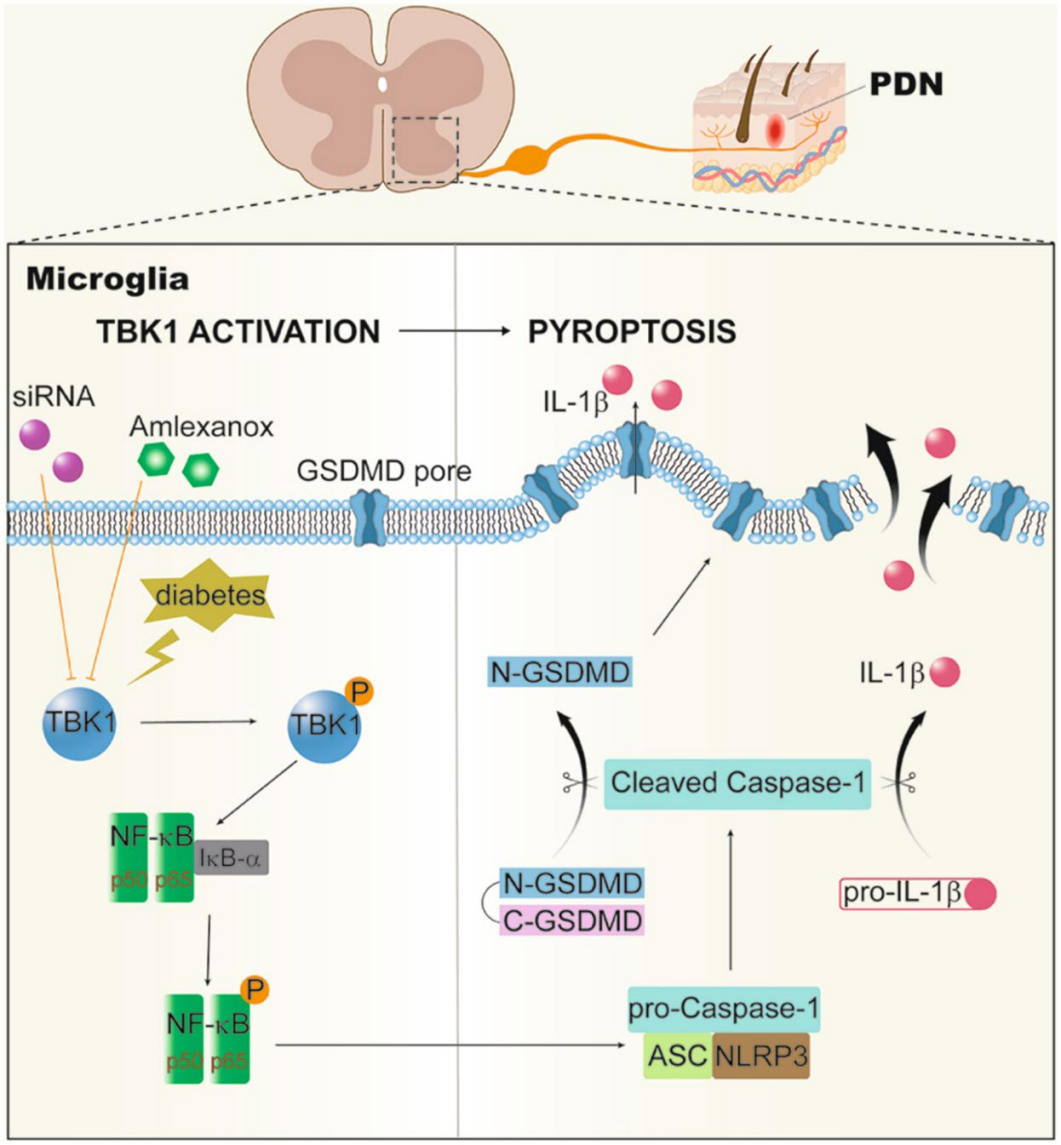
Schematic of Proposed Mechanisms

## Discussion

This study found that TBK1 was remarkably activated in a PDN mouse model and contributed to peripheral nerve injury and hyperalgesia. Intrathecal injection of TBK1-siRNA strikingly alleviated hyperalgesia via inhibiting NF-κb-related NLRP3 inflammasome activation and GSDMD-sparked microglia pyroptosis. Importantly, AMX systemic administration to diabetic db/db mice with PDN prevented and reversed peripheral neuropathy by reducing sciatic nerve damage, increasing intraepidermal nerve fibers, and improving blood perfusion of plantar skin. Together, our results indicated that TBK1 activation-regulated microglia pyroptosis in the SDH following PDN contributes to developing and maintaining neuropathic pain.

Previously, TBK1 garnered widespread attention owing to its immune and anti-tumor effects. Based on recent compelling evidence, TBK1 contributes to reducing inflammation and regulating energy metabolism [35]. However, our results suggested that inhibiting TBK1, whether using siRNA or AMX, could not improve hyperglycemia or obesity in db/db mice, a type of mutant mouse of the Lepr gene. The results revealed that TBK1 did not affect metabolic disorders in db/db mice, which might be related to the particularity of the model. We speculated that the effect of AMX on increasing energy consumption may not be sufficient to counteract the thermogenic defect and energy storage effect caused by leptin receptor deficiency in db/db mice, resulting in high blood glucose and body weight [36].

The canonical NF-κB signaling pathway has been widely reported in neuroinflammatory disease research. However, the relationship and physiological function of the noncanonical NF-κB signaling pathway in PDN are yet to be defined. Increasing evidence suggests that the noncanonical IKK protein TBK1 plays an activating role in regulating NF-κB. TBK1 requires scaffold protein assembly to effectively target its substrates, performed by TANK, NAP1, and SINTBAD [37]. Among them, TANK is a TRAF-binding protein, TBK1 can directly bind to its stimulating region, and TANK binds to TRAF2 to form a TBK1-TANK-TRAF2 complex, mediating IκB phosphorylation and activating NF-κB synergistically [38], involved in the inflammatory cascade in various tissue sites [9]. Our results suggested that NF-κB was activated in PDN mice, and TBK1 inhibition downregulated NF-κB activation, suppressing the noncanonical NF-κB pathway in PDN models. Luo et al. [33] reported that inhibiting the TLR4/NF-κB/NLRP3 signaling pathway regulated the microglial polarization to improve motor function in cerebral ischemia mice, demonstrating the interplay of NF-κB and NLRP3 inflammasome [24, 26]. Notably, our study findings validated pyroptosis in a mouse model of PDN and illustrated that inhibiting TBK1 affected the NLRP3 inflammasome and pyroptosis-related molecules, including caspase-1 p20, N-GSDMD, ASC, and cleaved IL-1β.

Pyroptosis is GSDMD-mediated programmed necrosis characterized by disruption of plasma membrane integrity, resulting in extracellular spillage of proinflammatory cytokines, such as IL-1β and IL-18. It has been reported that the upregulation of inflammatory mediators in cerebrospinal fluid was related to central sensitization [39], but our study mainly focused on the SDH related to sensory pathways, the expression levels of inflammatory mediators such as IL-1β in cerebrospinal fluid need to be further explored. Recent studies have reported that GSDMD is the critical executor of pyroptosis, and the N-GSDMD is a candidate for pyroptosis pore formation [19–26]. Therefore, this study analyzed the protein levels and cellular localization of GSDMD to explore the mediating role of pyroptosis in PDN.

Notably, elevated TBK1 was predominantly localized in the microglia of the SDH following PDN in diabetic db/db mice; the same cell localization has been reported in Parkinson’s disease model [40]. With in-depth research on pain, microglia are not only immune cells of the central nervous system but also participate in transmitting pain signals [41]. Previous research has established the role of microglial pyroptosis as a mediator of spinal cord injury [25] and the neuroinflammatory response following subarachnoid hemorrhage [26]. This study also found microglial pyroptosis in the SDH of db/db mice, and the use of caspase-1 inhibitor to directly inhibit the pyroptosis of microglia could alleviate PDN in db/db mice, demonstrating the importance of microglia pyroptosis in the PDN pathogenesis. This study illustrated that silencing TBK1 via TBK1-siRNA could alleviate microglial pyroptosis in SDH of db/db mice, thereby alleviating hyperalgesia.

Interestingly, elevated expression of c-Fos, a marker of pain circuitry, could occur when neurons are stimulated, which may indicate neuronal abnormalities in PDN mice. We discovered that intrathecal injection of TBK1-siRNA could save DRG neurons’ ability to extend axons in vitro, improving the stimulatory activity and pyroptosis in DRG neurons but exhibiting limited effect on other peripheral nerves, which may be related to the limited range of action of siRNA intrathecal injection. The efficacy of systemic administration of AMX on peripheral nerves proved this hypothesis. These results suggested that TBK1 may play a role in the peripheral nervous system of PDN mice, such as DRG, and the related mechanism needs to be further explored. AMX systemic administration was found to enlarge the impact on the peripheral nerve. AMX is an azoxanthone drug used for treating mouth aphthous ulcers, asthma, and allergic rhinitis; however, more and more scholars are focusing on AMX from tumors to metabolic diseases [16, 35, 42]. Loss of unmyelinated and myelinated fibers of the peripheral nerve, axonal degeneration, and segmental demyelination are recognized histopathological criteria for progressive PDN [43]. The sciatic nerve of db/db mice was reported to have decreased myelin thickness [44] and axonal atrophy; however, no sign of segmental demyelination was found even in the late stage of PDN [45], consistent with our research results. Baum et al. [46] observed infiltration of macrophages and T cells in the sciatic nerve in T1DM model rats, which increased by 25–50% compared to non-diabetic rats. Tian et al. [47] reported overexpression of proinflammatory cytokines and infiltration of M1 macrophages in the sciatic nerve of T2DM-related PDN mice. We speculated that AMX downregulated sciatic nerve inflammation in PDN mice and thus improved nerve injury.

PDN involves small and large nerve fibers, while in the early stage, it primarily involves small nerve fibers, which mediates sensory dysfunction. The quantification of intraepidermal nerve fiber is closely related to the threshold of mechanical allodynia and thermal hyperalgesia. A report about a plantar biopsy in patients with peripheral neuropathy stated that the decrease in the threshold of thermal hyperalgesia was related to the reduced IENFD, which often occurred earlier than the decrease in the threshold of thermal hyperalgesia [48]. The current study demonstrated that AMX could improve the decreased IENFD significantly in db/db mice and delay the deterioration of blood perfusion of plantar skin. Diabetes is often accompanied by peripheral vascular lesions, including microvascular disorders of basement membrane thickening and lumen stenosis, and similar lesions also exist in endomembrane microvessels [49]. The improved hyperalgesia in db/db mice following AMX treatment may be due to the reduced destruction of the intimal capillary-nerve barrier and a certain protective effect on intraepidermal nerve fiber.

## Conclusions

Overall, our data demonstrated that the inhibition of TBK1 could downregulate the level of inflammation in the SDH of PDN mice, inhibiting microglial pyroptosis, alleviating peripheral nerve injury, and finally reducing hyperalgesia.

### Electronic supplementary material

Below is the link to the electronic supplementary material.

Supplementary Material 1

Supplementary Material 2

Supplementary Material 3

Supplementary Material 4

## Data Availability

All data generated during this study are included either in this article or in the supplementary information files.

## Abbreviations

AMX: Amlexanox
cGAS: BCyclic GMP-AMP synthase
DMSO: Dimethyl sulfoxide
DRG: Dorsal root ganglia
GSDMD: Gasdermin D
IENFD: Intraepidermal nerve fiber density
IKK: IkB kinase
IRF3: Interferon regulatory factor 3
NF-κb: Noncanonical nuclear factor κB
N-GSDMD: N-terminal domain of GSDMD
NLRP3: Nod-like receptor family pyrin domain-containing 3
PDN: Painful diabetic neuropathy
PWL: Paw withdrawal latency
PWT: Paw withdrawal threshold
ROI: Region of interest
PU: Perfusion units
SDH: Spinal dorsal horn
SPF: Specific pathogen-free
STING: Stimulator of interferon gene
STZ: Streptozotocin
T1DM: Type 1 diabetes mellitus
T2DM: Type 2 diabetes mellitus
TBK1: TANK-binding kinase 1
