# Supplementary figures and images for: Targeting TANK-binding kinase 1 attenuates painful diabetic neuropathy via inhibiting microglia pyroptosis
Source: Cell Commun Signal. 2024 Jul 19;22:368. doi: 10.1186/s12964-024-01723-6 (PMC11264750; doi:10.1186/s12964-024-01723-6)

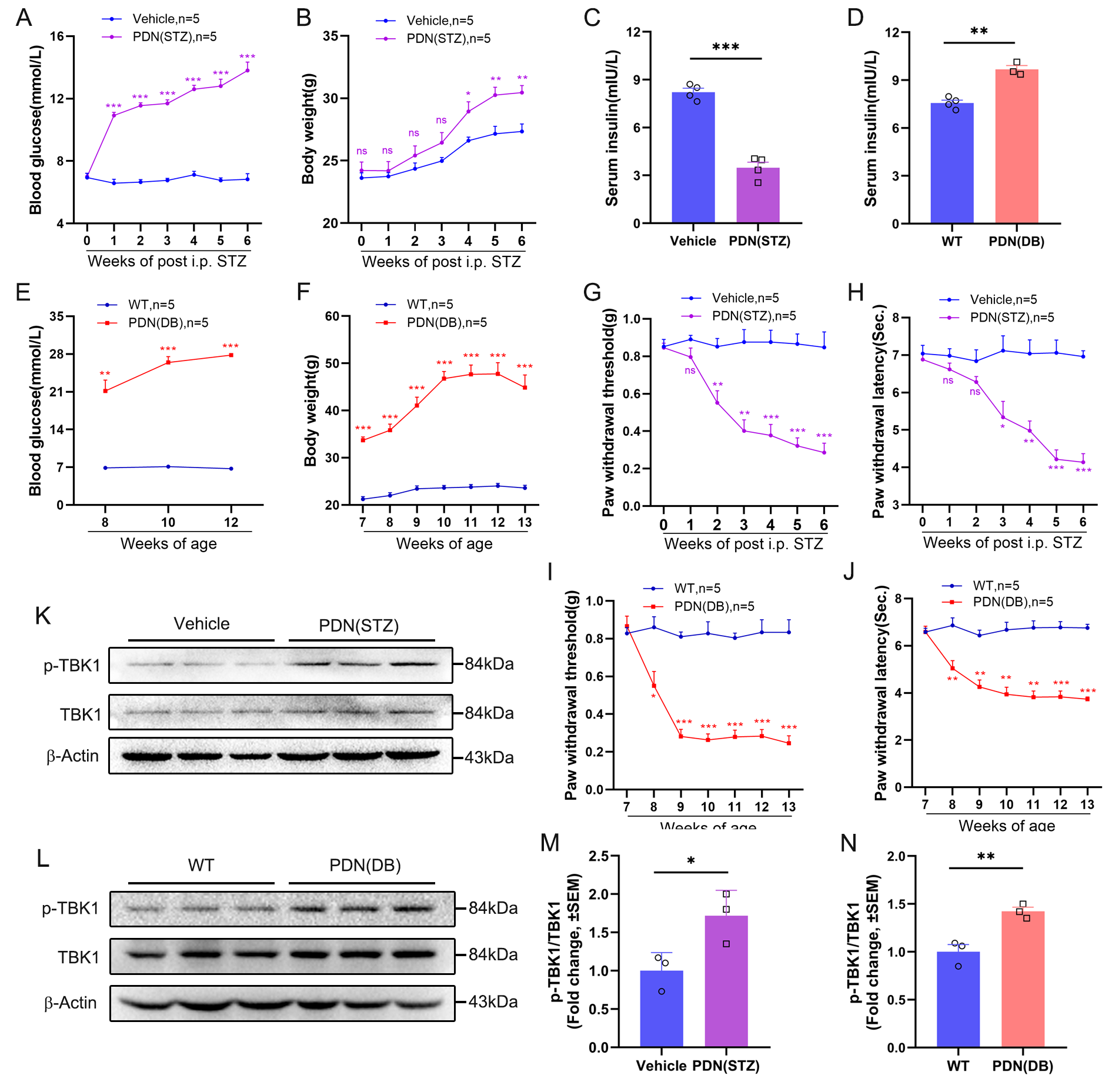

Supplement: Supplementary file 1 — Supplementary Material 1 [file 12964_2024_1723_MOESM1_ESM.tif]

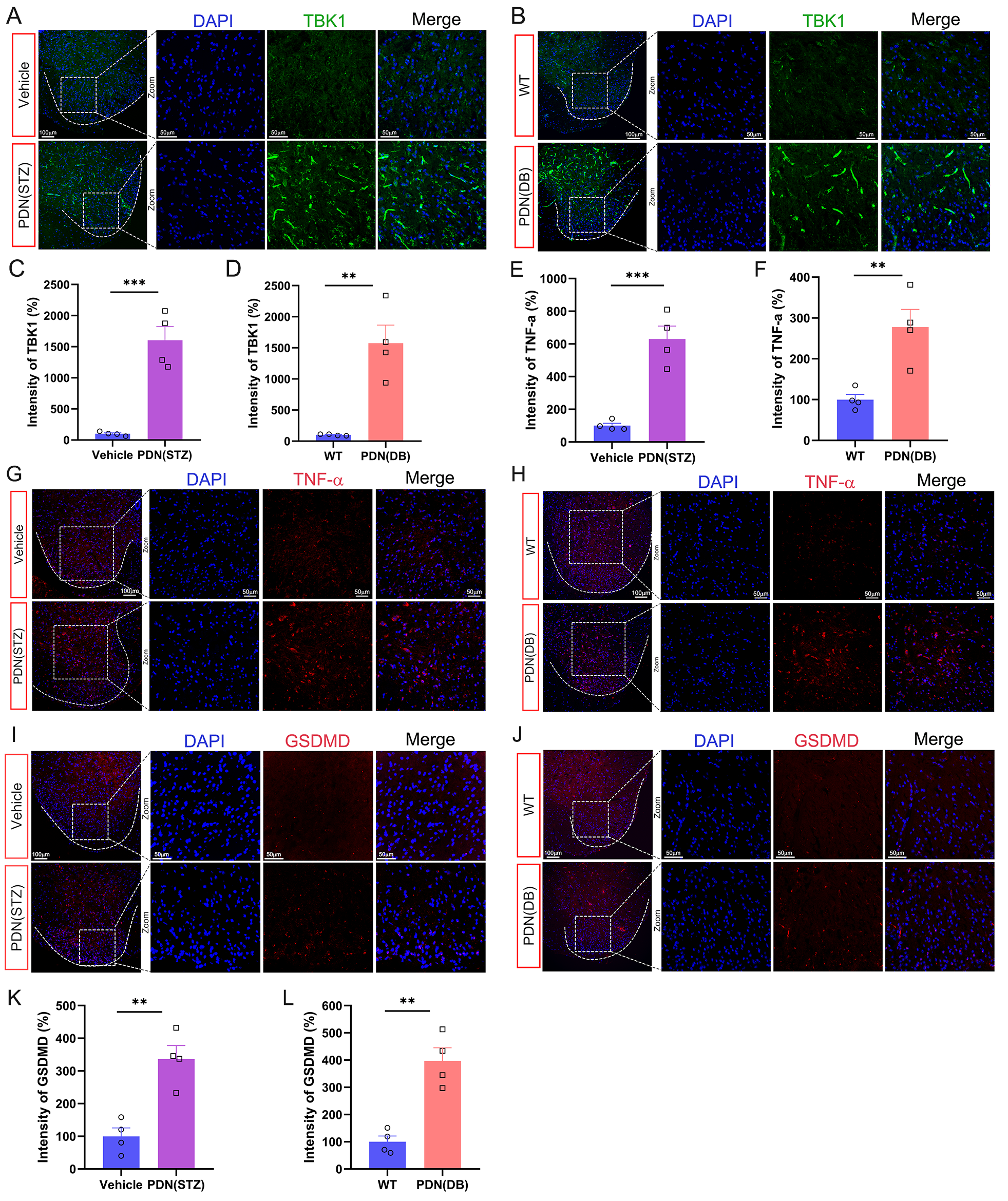

Supplement: Supplementary file 2 — Supplementary Material 2 [file 12964_2024_1723_MOESM2_ESM.tif]

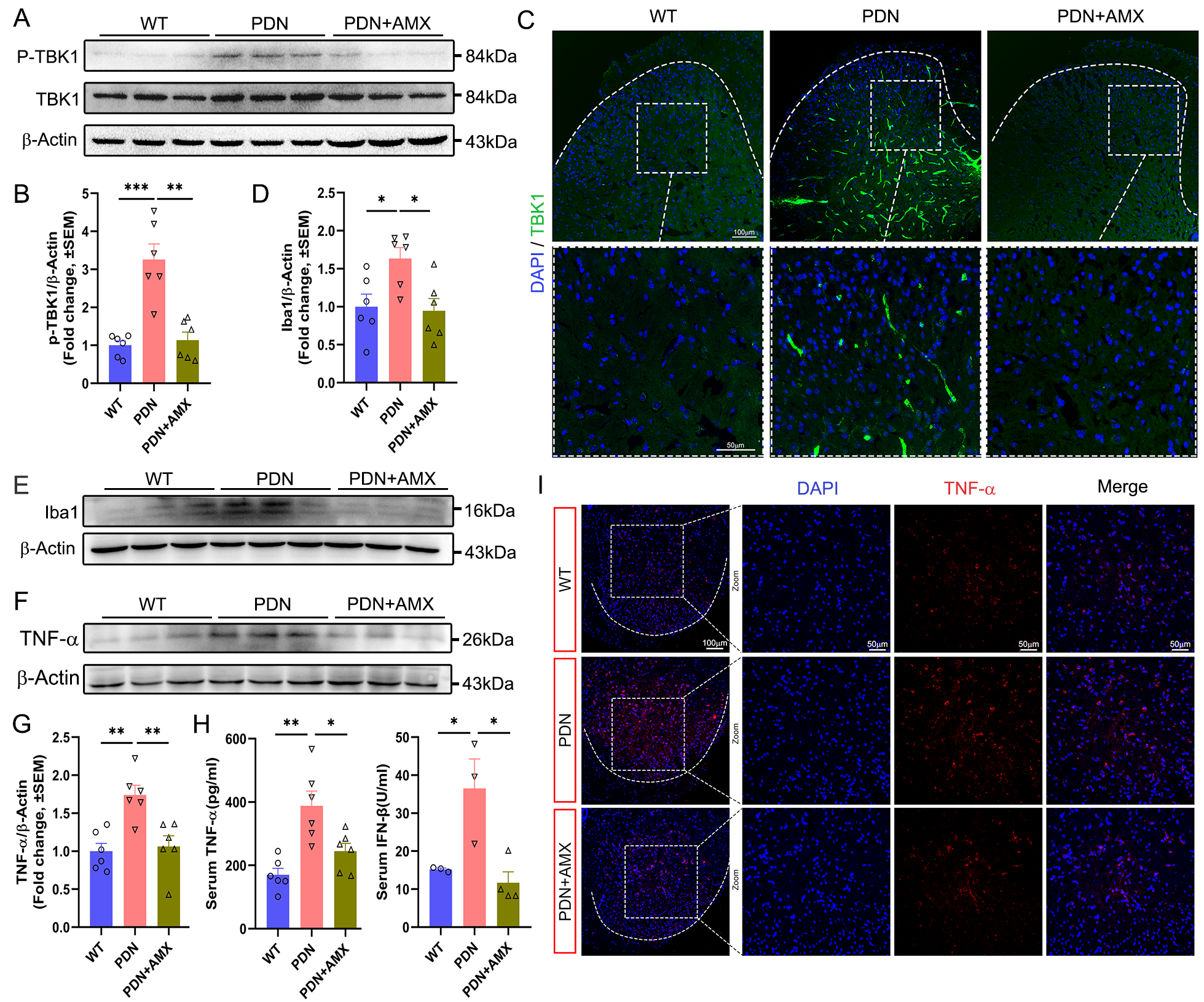

Supplement: Supplementary file 3 — Supplementary Material 3 [file 12964_2024_1723_MOESM3_ESM.tif]
